# *Schistosoma haematobium* Treatment in 1–5 Year Old Children: Safety and Efficacy of the Antihelminthic Drug Praziquantel

**DOI:** 10.1371/journal.pntd.0001143

**Published:** 2011-05-17

**Authors:** Francisca Mutapi, Nadine Rujeni, Claire Bourke, Kate Mitchell, Laura Appleby, Norman Nausch, Nicholas Midzi, Takafira Mduluza

**Affiliations:** 1 Ashworth Laboratories, Institute of Immunology & Infection Research, School of Biological Sciences, University of Edinburgh, Edinburgh, United Kingdom; 2 National Institute of Health Research, Causeway, Harare, Zimbabwe; 3 Department of Biochemistry, University of Zimbabwe, Mount Pleasant, Harare, Zimbabwe; Hospital Universitário, Brazil

## Abstract

**Background:**

Morbidity due to schistosomiasis is currently controlled by treatment of schistosome infected people with the antihelminthic drug praziquantel (PZQ). Children aged up to 5 years are currently excluded from schistosome control programmes largely due to the lack of PZQ safety data in this age group. This study investigated the safety and efficacy of PZQ treatment in such children.

**Methods:**

Zimbabwean children aged 1–5 years (n = 104) were treated with PZQ tablets and side effects were assessed by questionnaire administered to their caregivers within 24 hours of taking PZQ. Treatment efficacy was determined 6 weeks after PZQ administration through schistosome egg counts in urine. The change in infection levels in the children 1–5 years old (n = 100) was compared to that in 6–10 year old children (n = 435).

**Principal Findings:**

Pre-treatment *S. haematobium* infection intensity in 1–5 year olds was 14.6 eggs/10 ml urine and prevalence was 21%. Of the 104 children, 3.8% reported side effects within 24 hours of taking PZQ treatment. These were stomach ache, loss of appetite, lethargy and inflammation of the face and body. PZQ treatment significantly reduced schistosome infection levels in 1–5 year olds with an egg reduction rate (ERR) of 99% and cure rate (CR) of 92%. This was comparable to the efficacy of praziquantel in 6–10 year olds where ERR was 96% and CR was 67%.

**Interpretation/Significance:**

PZQ treatment is as safe and efficacious in children aged 1–5 years as it is in older children aged 6–10 years in whom PZQ is the drug of choice for control of schistosome infections.

## Author Summary

Urogenital schistosomiasis is an important, but neglected, infectious disease affecting over 100 million people, mainly in Africa. Children carry the heaviest burden of infection with children as young as 1 year old showing signs of infection. Children aged 5 years and below are currently excluded from schistosome control programmes for several reasons, including operational difficulties associated with accessing preschool children, misconceptions about their level of exposure to infective water and lack of safety data on the drug of choice for schistosome control, praziquantel, in children aged 5 years and below. This study was one of a small number of studies recently funded by the World Health Organization to investigate the need for praziquantel treatment in preschool children (aged 1–5 years) and to subsequently assess the safety and efficacy of the drug praziquantel in this age group. This study confirmed that preschool children carry significant levels of schistosome infection, exceeding those carried by their parents/guardians, highlighting the urgent need for their immediate inclusion in schistosome control programmes. The study also showed that praziquantel treatment is as safe and efficacious in children aged 1–5 years as it is in older children aged 6–10 years who are currently the target for mass drug administration.

## Introduction

Classified among the neglected tropical diseases, urogenital schistosomiasis (bilharzia), remains one of the most prevalent parasitic diseases in the tropical and subtropical countries, constituting a major public health problem. The disease is caused by the helminth parasite *Schistosoma haematobium* and is the most prevalent form of schistosomiasis in Africa and the Middle East affecting approximately 107 million people. In affected populations, children carry the heaviest burden of infection [1], [2]n and in young children, urogenital schistosomiasis causes haematuria, dysurea, nutritional deficiencies, anaemia, growth retardation, decreased physical performance and impaired memory and cognition [3], [4], [5], [6], [7].

Control of schistosome infections is through treatment of infected people with a single dose of the anti-helminth drug praziquantel which is safe, highly efficacious, cheap (costing less than US$0.50/dose) and can reverse schistosome-related morbidity particularly in the early stages of disease progression [8]. Current schistosome control programmes advocated by the World Health Assembly in 2001 through resolution 54·19's recommend regular de-worming of school aged children at risk of infection (http://www.who.int/infpr-2001/en/pr2001WHA-6.html), but exclude pre-school children i.e. children aged 5 years and below due to the belief that these children are not sufficiently exposed to infective water to experience high infection rates [9] which would lead to the clinical manifestation of disease and the lack of safety data on praziquantel in this age group [10]. However, several studies have now shown prevalent schistosome infection (as much as 100% in some areas) and morbidity of African children below the age of 5 [11], [12], [13], [14]. Thus these children are both at risk of infection and a potential reservoir for the parasite in communities successfully targeted by mass anti-helminthic treatment. These findings have led to a growing number of calls to include pre-school children and infants in schistosome-control programmes [9], [11], [13], [15]. We have previously assessed the side effects reported following PZQ treatment of Zimbabwean school children exposed to *S. haematobium* aged 6 years and above [16]. However, there have not been any studies on the safety of PZQ treatment of *S. haematobium* infections in children aged below 5 years of age. Furthermore, given that PZQ works synergistically with the host immune system to kill the worms, the efficacy of the drug in young children whose immune system might differ from older children may be lower. Therefore the present study assesses the safety of PZQ- treatment in 1–5 year old children in Zimbabwe naturally exposed to *S. haematobium* infection. It also compares efficacy rates in the 1–5 year old to those in 6–10 year old children testing the hypotheses that (1) PZQ treatment reduces infection levels and (2) PZQ efficacy rates in 1–5 year olds are similar to those in 6–10 year olds.

## Materials and Methods

### Study area and population

The study was conducted in 3 villages, Magaya, Chipinda and Chitate, in the Mashonaland East Province of Zimbabwe (31°30′E; 17°45′S) where *S. haematobium* is endemic. Similar to other rural areas in Zimbabwe, there is a low prevalence of *Schistosoma mansoni* and soil transmitted helminths in this region [2], [17], [18]. There had not been any schistosome control or research programmes in the villages prior to this study. The participants are involved in ongoing studies on the control and immuno-epidemiology of human schistosomiasis. The main activity in these villages is subsistence farming and human water contact is frequent with at least 4 contacts/person/week (assessed by questionnaire) due to insufficient safe water and sanitation facilities. The questionnaire studies also confirmed that older children are exposed to infective water actively while younger children are also exposed passively when accompanying parents to river sites or through river water bought back home [9] as indicted by questionnaire responses of their parents/guardians.

### Ethical statement

Permission to conduct the study in the region was obtained from the Provincial Medical Director. Institutional and ethical approval was received from the University of Zimbabwe and the Medical Research Council of Zimbabwe respectively. In addition, the study received ethical approval from the World Health Organization's Research Ethics Review Committee. At the beginning of the study, parents and guardians of participating children had the aims and procedures of the project explained fully in the local language, Shona, and written consent was obtained from participants' parents/guardians before enrolment into the study. After collection of all samples, all compliant participants (children under 5 years of age, older children (6 years and above) and all parents/guardians) were offered anti-helminthic treatment with the recommended dose of praziquantel (40 mg/kg of body weight).

### Case-history questionnaires

At enrolment into the study, the parents/guardians of the children (1–5 years old) were asked 30 questions on behalf of the child recording demographic information, general socio-economic indicators, access to health care, general self-reported health conditions, water contact behaviours and general awareness and knowledge on schistosome infection. Questionnaires were administered in Shona by a trained team member. The questionnaire responses on the current health status of the child and the clinical assessment of the nursing staff informed on the suitability of the child to partake in the study.

### Sample collection, PZQ treatment and assessment of side effects

Stool and urine specimens were collected from each participant on 3 consecutive days and processed using microscopic examination of urine samples for *S. haematobium* following urine filtration [19], and microscopic examination of stool samples for *S. mansoni* and geo-helminths following the Kato Katz technique [20]. The formol-ether concentration method was performed as previously described [2], [21] on a random sample of 25% of the stool samples to confirm results obtained by the Kato-Katz technique. For infants, samples were collected overnight if it was not possible to collect a sample on the spot. After collection of the parasitology samples, participants (children and their guardians/parents) were offered treatment with the standard dose of praziquantel, i.e. 40 mg/kg body weight. Dose was determined for all children by weighing and the weight measure was rounded off to the nearest kg to determine the dose for each child and where necessary, tablets were broken in half or quarters to make up the appropriate dose. Irrespective of infection status, all compliant participants (parents/guardians and children) were treated with praziquantel tablets obtained from the IDA Foundation (http://www.idafoundation.org/, catalogue number 13200; 6600 mg/tablet). This was in keeping with mass drug administration practices during the investigative epidemiological surveys. For children aged 5 years and below, tablets were crushed using spoons and administered by the parent/guardian under supervision of one of the research teams. The tablets were taken with juice. Acceptability of the tablet was assessed directly during PZQ drug administration. A child would be recorded as not accepting drug administration if he/she spat, choked or vomited the drug. All treated people were given bread to eat after antihelminthic treatment. Parents/guardians were encouraged to remain at the treatment centre for 1 hour after administering the medication to assess if the PZQ treatment had immediate side effects and to determine whether the medication was lost through vomiting by the participants. Parents/guardians reported back 24 hours after PZQ treatment (preferably with the child) to answer a questionnaire with 9 questions on side effects following PZQ administration (i.e. not present when PZQ was initially administered). Participants who would not accept treatment on religious grounds or were absent from school on treatment days but wished to remain part of the study cohort effectively became untreated controls.

### Inclusion and exclusion criteria

In order to be included in the PZQ safety study, participants had to meet all the following criteria: 1) be aged 1–5 years at recruitment, 2) had been resident in the study area since birth, 3) had provided at least two urine and two stool samples on consecutive days, 4) be negative for intestinal helminths and *S. mansoni* (no one was excluded on this criteria as everyone was negative for these infections as is reported in other parts of Zimbabwe [22]), 5) have successfully taken the PZQ tablets prescribed to them and, 6) parents/guardians had provided answers to the 24 hour post-treatment side effects questionnaire. 104 participants met these criteria and were included in the PZQ safety study. To be included in the treatment efficacy study, the children had to meet criteria 2–4 above. In addition they had to have been offered PZQ treatment if present on treatment days and had provided at least 2 urine and 2 stool samples on consecutive days, 6 weeks after treatment with PZQ. For both studies, children were excluded if the parents/guardians/the child themselves (in the case of older children) reported a pre-existing illness or if they were suffering from a fever as assessed by clinical examination by the nursing staff. 100 children aged 1–5 years of age met this criteria (72 treated with PZQ, 28 untreated) and 435 children aged 6–10 years old (355 treated with PZQ and 80 untreated). The larger sample size of 6–10 year olds reflects the school-based recruitment design of the study.

### Statistical analysis

Statistical tests using the statistical package in PASW (formerly SPSS) were used to test two hypotheses (1) PZQ treatment significantly reduces infection levels and (2) PZQ efficacy rates in 1–5 year olds are similar to those in 6–10 year olds. The effect of treatment on infection prevalence levels for the whole study group was tested using a logistic regression approach with variables selected using the forward stepwise conditional method [23]. The dependent variable was infection status 6 weeks after treatment (infected vs. uninfected) while the independent variables were pre-treatment infection intensity (log_10_(x+1) transformed), sex (male vs. female), age group (group 1 (1–5 years old) vs. group 2 (6–10 years old)), treatment status (PZQ treated vs. untreated) and the interaction between age group and treatment status. The model passed the Hosmer and Lemenshaw goodness of fit of the test [23]. Infection prevalences between age groups and infection status were compared using a one-tailed chi-squared test with 95% CI calculated using a Binomial distribution.

Infection intensity was compared using repeated measures ANOVA. For this analysis, the dependent variable infection intensity was log_10_(x+1) transformed, the independent variables which were all categorical were sex (male, female) age group (two groups, group 1 = 1–5 years, group 2 = 6–10 years), treatment (1 = untreated, 2 = PZQ treated). Potential confounding effects of sex and village were allowed for by using sequential sums of squares with these two variables entered first and second in the model. The interaction between age-group and treatment was also tested to determine if the effect of treatment varied depending on age group. Test statistics were taken as significant at p≤0.05. Post-hoc paired T-tests were conducted to determine differences in infection levels between the time points (pre-treatment vs. 6 weeks post-treatment).

## Results

### Study area infection intensity

**Figure 1:**
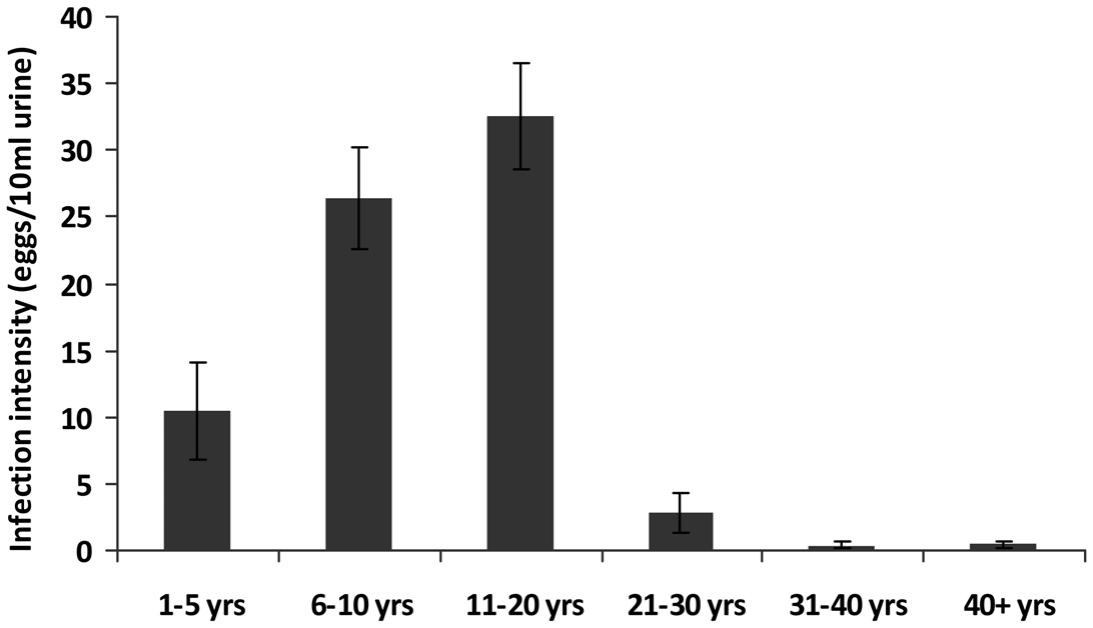
*Schistosoma haematobium* infection intensity in the study villages. Arithmetic mean of schistosome infection intensity for each age group. Bars represent standard error of the mean.

**Figure 2:**
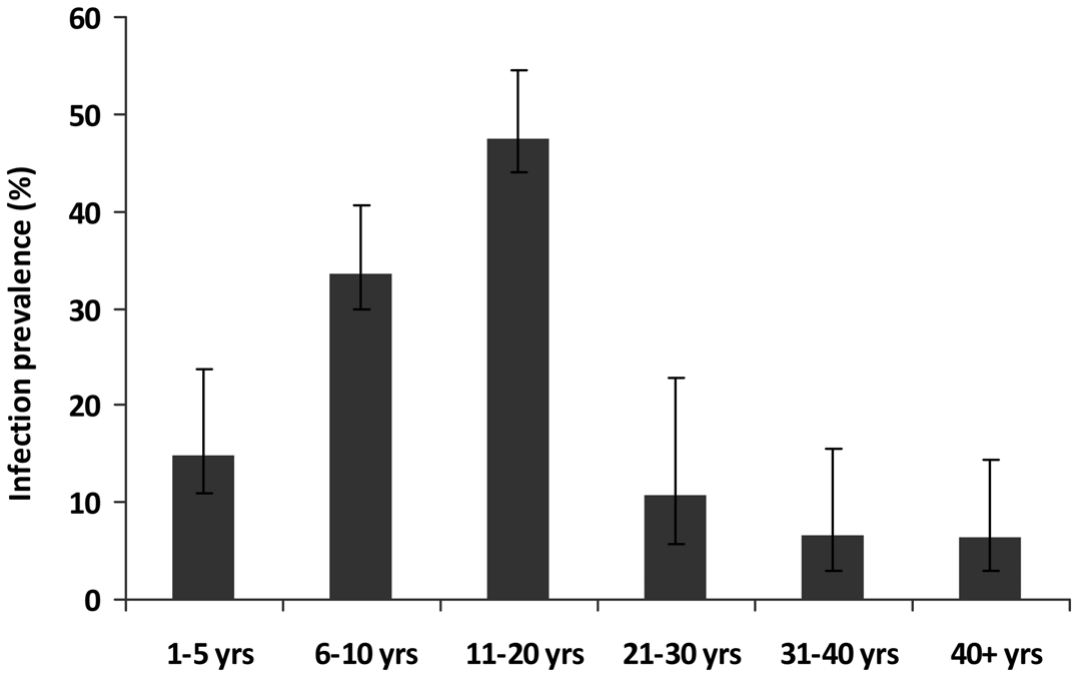
*Schistosoma haematobium* infection prevalence in the study villages. Infection prevalence for each age group. Bars represent 95% CI.

The children came from 3 villages endemic for *S. haematobium* infection; an initial survey of 1980 permanent residents of the study villages aged 1–80 years showed an overall infection prevalence of 30.5% and arithmetic mean infection intensity of 21.1 eggs/10 ml urine (SEM = 1.9 and range 0–1000 eggs/10 ml urine). Infection prevalence and intensity followed a convex age-infection profile with infection levels rising to peak in people aged 11 to 20 years old and infection intensity declining faster than infection prevalence thereafter (Figures 1 and 2). Schistosome infection levels in 1–5 year olds in the population were higher than in adults aged 21 years and above.

### Safety of PZQ in 1–5 year olds

PZQ drug dose was determined by weight which ranged from 8 kg to 24 kg in the 1–5 year olds (mean 16.7 kg, standard error of the mean = 0.46). During PZQ administration, there was only one record of failure to accept the drug, where the child vomited the tablets. There were no other side-effects directly related to tablet administration. 104 children were treated with PZQ and their parents completed side effects questionnaires 24 hours after the successful administration of PZQ tablets. *S. haematobium* eggs were detected in urine samples from 15 of these children. Side effects arising within 24 hours of treatment were reported in 4 children aged 3–5 years old, none egg positive for *S. haematobium* infection. The side effects reported were diarrhoea (3 children), lethargy (2 children), inflammation of the body and face (1 child, this has subsided by bedtime and was confirmed resolved by the nursing staff at the 24 hour examination), and loss of appetite (1 child).

### Treatment efficacy

**Figure 3:**
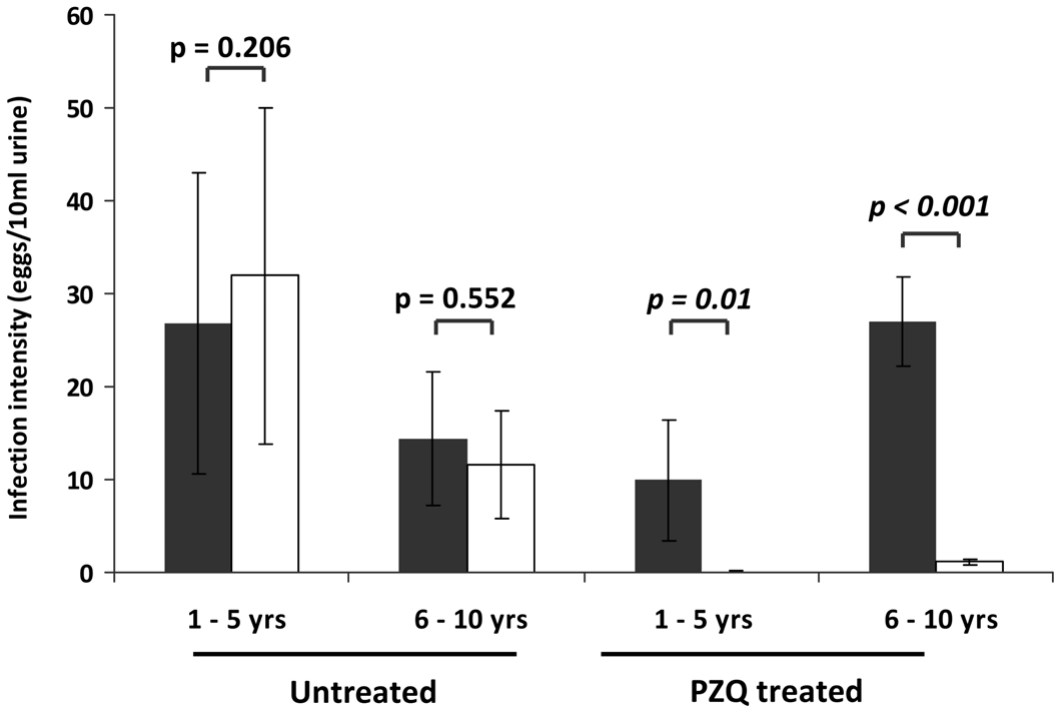
Praziquantel efficacy in reducing infection intensity in different age groups. Comparison of treatment efficacy in 1–5 year old children vs. 6–10 year old children. Infection intensity before treatment is shown in black and those 6 weeks after treatment are shown in white Bars represent standard error of the mean. Test-statistic values are from paired T tests.

535 children were followed-up at the two time points to determine the efficacy of PZQ treatment 6 weeks post treatment and test whether PZQ efficacy was age-dependent. Pre-treatment schistosome infection levels are shown in Table 1 by age group and by treatment status. A more detailed breakdown of pre-treatment infection distribution in the 100 children aged 1–5 years is given in Table 2. There was a significant reduction in *S. haematobium* infection intensity in children receiving PZQ treatment compared to untreated children in both age groups (F = 15.165, df = 1,528, p<0.001) as shown in Figure 3. In treated 1–5 year olds, infection intensity declined from a mean of 9.9 to 0.1 eggs/10 ml urine, giving an egg reduction rate of 99%. In untreated children, mean infection intensity increased from 26.8 to 32.0 eggs/10 ml urine, with an egg reduction rate of 19%. In treated 6–10 year olds, infection intensity declined from 27.0 to 1.1 eggs/10 ml urine (egg reduction rate = 96%) compared to the change in infection intensity in untreated children from 14.1 to 11.6 eggs/10 ml urine (egg reduction rate = 19%). The statistical analyses also showed that there was no significant interaction between treatment and age group (F = 0.563, df = 1,528, p = 0.45) indicating that there was no significant difference in the efficacy of PZQ treatment in 1–5 year olds compared to 6–10 year olds.

**Table 1:** Distribution of infection intensity and prevalence in both age groups (1–5 years old) and (6–10 years old) in the study population.

Age (years)	PZQ infection status	Sample size	Mean infection intensity*	SEM**	Min infection intensity	Max infection intensity	Infection prevalence (%)
**1–5**	Untreated	28	26.8	16.1	0	380	29
	PZQ–treated	72	9.9	6.4	0	458	18
	Total	100	14.6	6.5	0	458	21
**6–10**	Untreated	80	14.4	7.1	0	502	18
	PZQ–treated	355	27.0	4.8	0	878	38
	Total	435	24.7	4.1	0	878	34
**Total**	Untreated	108	17.6	6.7	0	502	20
	PZQ–treated	427	24.1	4.1	0	878	35
	Total	535	22.8	3.6	0	878	32

*Arithmetic mean, units = eggs/10 ml urine;

**Standard Error of the Mean.

**Table 2:** Distribution of infection intensity and prevalence in 1–5 year old children in the study population.

Age (years)	Sample size	Mean infection intensity*	SEM**	Infection prevalence (%)
**1.0–3**	13	0.2	0.2	8
**3.1–4**	24	22.4	19.1	17
**4.1–5**	63	14.7	7.3	25
**Total**	100	14.6	6.5	21

*Arithmetic mean, units = eggs/10 ml urine;

**Standard Error of the Mean.

**Figure 4:**
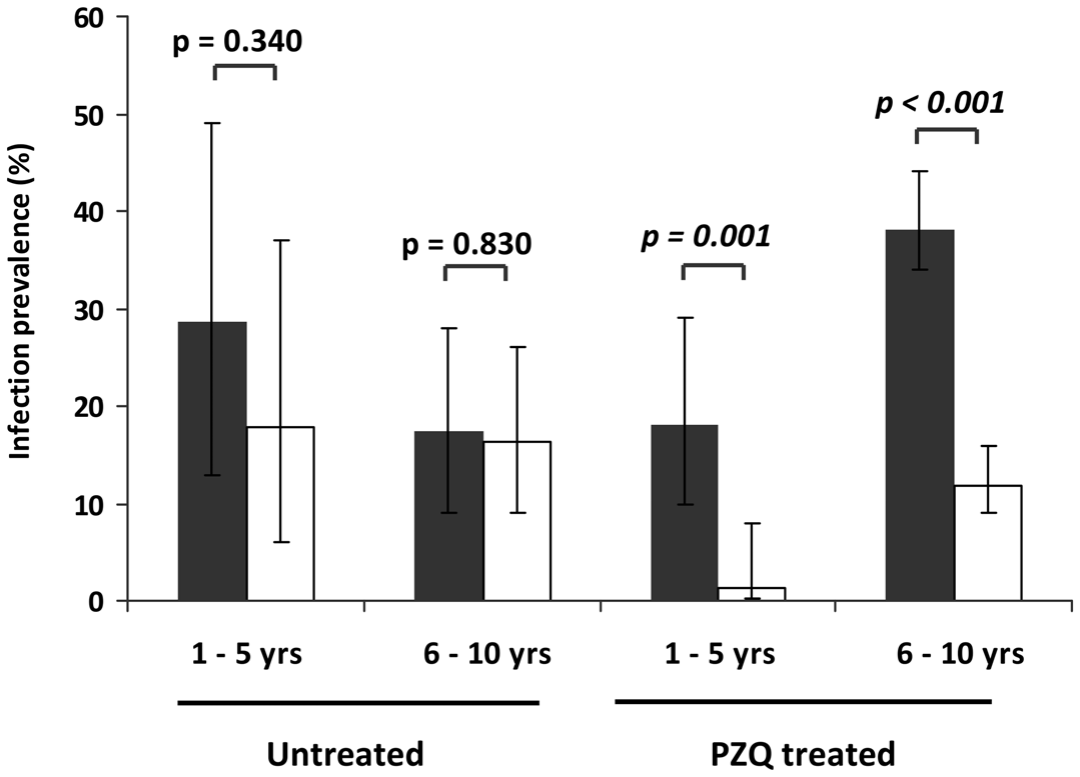
Praziquantel efficacy in reducing infection prevalence in different age groups. Comparison of PZQ treatment between 1–5 year old children vs. 6–10 year old children. Infection prevalence before treatment is shown in black and those 6 weeks after treatment are shown in white. Bars represent 95% CI. Test-statistic values are from χ2 tests.

Infection prevalence followed similar patterns with prevalences in treated children in both age groups declining significantly compared to untreated children in both age groups (Wald = 5.68, df = 1, p = 0.017) (Figure 4). In untreated children prevalences declined from 28.5% to 17.9% in 1–5 year olds (χ2 = 0.90, df = 1, p = 0.34) and 17.5% to16.5% in 6–10 year olds (χ2 = 0.05, df = 1, p = 0.83), giving cure rates of 38% and 7% respectively. In treated children prevalences fell from 18.0% to 1.4% in 1–5 year olds (χ2 = 11.4, df = 1, p = 0.001) and 38.0% to 11.8% in 6–10 year olds (χ2 = 65,1, df = 1, p<0.001) giving cure rates of 92% and 67% respectively. This difference in cure rates in treated children was not statistically significantly different between the age groups as the interaction between treatment and age-group was not significant.

## Discussion

Despite the public health prominence of schistosomiasis in Africa and the availability of a cheap and efficacious drug to treat infected people, fewer than 5% of the infected population is receiving treatment [10]. Included in the group of affected people currently not receiving treatment, are children aged 5 years and below. Current mass drug administration (MDA) schistosome control programmes exclude children aged 5 years and below for several reasons including (1) practicality; mass helminth control programmes in Africa are largely school based so as to take advantage of existing infrastructure serving an accessible population. These children tend to be aged 6 years and above which means non-enrolled children below the age of 6 are excluded from such programmes; (2) misconceptions about the levels of infection in this age group and (3) lack of safety data in this age group. This study showed that children aged 5 years and below carry infection levels higher than those of their parents/guardians i.e. people aged 21 and above, who are currently eligible for inclusion in MDA programmes). Furthermore, whilst it is known that schistosome infection intensity and morbidity are aggregated in school children, current estimates predict that pre-school children, who are relatively under-represented in field studies, also bear a significant urogenital disease burden in sub-Saharan Africa [24] Therefore, based on infection levels in these children the present study adds field data to support the growing calls for inclusion of children under 5 in schistosome control programmes [9], [11], [13], [15].

Although PZQ can be prescribed on a case-by-case basis in young children, there have not been studies on the safety of PZQ treatment of *S. haematobium* infection in children under 5 years of age with a view to include them in MDA programmes. In this study four out of 104 children (3.8%) reported mild side effects (headache, loss of appetite, stomach ache and general weakness) occurring within 24 hours after taking praziquantel tablets which is less than what we have reported in older (primary school) children [16] and that reported from *S. mansoni* studies in endemic [25] and epidemic [26] areas. There are likely to be biases in parent/guardian reporting side effects on behalf of the child, but this approach has recently been used successfully in studies of the safety of PZQ treatment in 0–5 year olds exposed to *S. mansoni* infections which reported higher percentages of side effects than those reported in the present study. The low percentage of children suffering side effects in the present study may be related to the low levels of infection they were carrying since previous studies show that the frequency and severity of side effects is proportional to the intensity of schistosome infection [25], [26]. Both the safety and efficacy studies presented here included egg negative and egg positive children since microscopic examination of urine and stool samples for parasite eggs are currently the ‘gold’ standard field method of diagnosing schistosome infections, but the procedures can miss light, single sex or pre-patent infections. The lower levels of side effects reported here compared to those reported in *S. mansoni* infections may also be related to differences between the parasites. Olds et al reported different levels of PZQ- related side effects between children infected with *S. mansoni* and *S. haematobium* [27].

PZQ treatment was efficacious, significantly reducing infection intensity and prevalence in treated children compared to untreated children. There were changes in infection levels in the untreated group of children in the 6 weeks between the recruitment and efficacy check surveys possibly reflecting both transmission related changes in infection levels as well as inaccuracies in the diagnosis. A number of studies have demonstrated that the schistosomicidal effect of praziquantel depends upon the immune status of the host [28], [29]. Our previous studies and those of others have indicated that acquired immunity against schistosomes develops slowly over several years [30], [31], [32], [33], [34], [35], [36], [37], [38]. Such results have often been taken as an indication that the immune responses of very young children may not have developed sufficiently to provide the synergy required for efficient praziquantel action. Therefore, PZQ treatment efficacy rates were compared between 1–5 year old children and 6–10 year old children. The egg reduction rates of 99% and 96% respectively were comparable between the two age groups while the cure rate of 92% in 1–5 year olds was higher than the 67% in 6–10 year olds, although this difference was not statistically significant.. Thus, the present results do not support the suggestion that the immune system in 1–5 year olds is not sufficiently developed to act synergistically with praziquantel rather, the results indicate that these children can be successfully cured of schistosome infections and argues for their inclusion in schistosome control programmes both to reduce schistosome infection and to induce earlier expression of protective responses which may help reduce morbidity in later life [34], [39]. The higher cure rates in the 1–5 year olds are likely to be related to the lower levels of infection they were carrying compared to the older children, an observation we have previously reported in older children [16] although the possibility of early re-infection in the older children which would mask cure rates, cannot be entirely ruled out.Both ERR and CR were within the values we and others have previously reported for *S. haematobium* infections [16], [40], [41], [42].

Taken together, the present study found that *S. haematobium* infection levels in 1–5 year olds exceeded those in adults aged 21 and above. Side effects following administration of PZQ were low suggesting that PZQ treatment was safe in the 1–5 year old children. PZQ treatment was also efficacious in this age group reducing infection levels and treatment efficacy was comparable to that in 6–10 year olds.

## References

[1] Gryseels B, de Vlas SJ (1996). Worm burdens in schistosome infections.. Parasitol Today.

[2] Midzi N, Sangweme D, Zinyowera S, Mapingure MP, Brouwer KC (2008). The burden of polyparasitism among primary schoolchildren in rural and farming areas in Zimbabwe.. Trans R Soc Trop Med Hyg.

[3] Stephenson LS, Latham MC, Kurz KM, Miller D, Kinoti SN (1985). Urinary iron loss and physical fitness of Kenyan children with urinary schistosomiasis.. Am J Trop Med Hyg.

[4] Stoltzfus RJ, Albonico M, Tielsch JM, Chwaya HM, Savioli L (1997). Linear growth retardation in Zanzibari school children.. J Nutr.

[5] Koukounari A, Gabrielli AF, Toure S, Bosque-Oliva E, Zhang Y (2007). *Schistosoma haematobium* infection and morbidity before and after large-scale administration of praziquantel in Burkina Faso.. J Infect Dis.

[6] Jukes MC, Nokes CA, Alcock KJ, Lambo JK, Kihamia C (2002). Heavy schistosomiasis associated with poor short-term memory and slower reaction times in Tanzanian schoolchildren.. Trop Med Int Health.

[7] Bhargava A, Jukes M, Lambo J, Kihamia CM, Lorri W (2003). Anthelmintic treatment improves the hemoglobin and serum ferritin concentrations of Tanzanian schoolchildren.. Food Nutr Bull.

[8] King CH (2006). Long-term outcomes of school-based treatment for control of urinary schistosomiasis: a review of experience in Coast Province, Kenya.. Mem Inst Oswaldo Cruz.

[9] Stothard JR, Gabrielli AF (2007). Schistosomiasis in African infants and preschool children: to treat or not to treat?. Trends Parasitol.

[10] Hotez PJ, Fenwick A (2009). Schistosomiasis in Africa: an emerging tragedy in our new global health decade.. PLoS Negl Trop Dis.

[11] Garba A, Barkire N, Djibo A, Lamine MS, Sofo B (2010). Schistosomiasis in infants and preschool-aged children: Infection in a single *Schistosoma haematobium* and a mixed *S. haematobium-S. mansoni* foci of Niger.. Acta Trop.

[12] Mafiana CF, Ekpo UF, Ojo DA (2003). Urinary schistosomiasis in preschool children in settlements around Oyan Reservoir in Ogun State, Nigeria: implications for control.. Trop Med Int Health.

[13] Sousa-Figueiredo JC, Basanez MG, Mgeni AF, Khamis IS, Rollinson D (2008). A parasitological survey, in rural Zanzibar, of pre-school children and their mothers for urinary schistosomiasis, soil-transmitted helminthiases and malaria, with observations on the prevalence of anaemia.. Ann Trop Med Parasitol.

[14] Uneke JC, Egede MU (2009). Impact of urinary schistosomiasis on nutritional status of school children in south-eastern Nigeria.. Internet J Health.

[15] Sousa-Figueiredo JC, Pleasant J, Day M, Betson M, Rollinson D (2010). Treatment of intestinal schistosomiasis in Ugandan preschool children: best diagnosis, treatment efficacy and side-effects, and an extended praziquantel dosing pole.. Intern Health.

[16] Midzi N, Sangweme D, Zinyowera S, Mapingure MP, Brouwer KC (2008). Efficacy and side effects of praziquantel treatment against *Schistosoma haematobium* infection among primary school children in Zimbabwe.. Trans R Soc Trop Med Hyg.

[17] Milner TM, Reilly LJ, Nausch N, Midzi N, Mduluza T (2010). Circulating cytokine levels and antibody responses to human *Schistosoma haematobium*: IL-5 and IL-10 levels depend upon age and infection status.. Parasite Immunol.

[18] Ndhlovu P, Chimbari M, Ndmba J, Chandiwana SK (1996). 1992 National Schistosomiasis Survey: Blair Research Institute Report for Zimbabwe.. Blair Research Institute.

[19] Mott KE (1983). A reusable polyamide filter for diagnosis of *S. haematobium* infection by urine filtration.. Bull Soc Pathol Exot.

[20] Katz N, Chaves A, Pellegrino J (1972). A simple device for quantitative stool thick smear technique in schistosomiasis mansoni.. Rev Instit Med Trop Sao Paulo.

[21] Cheesbrough M (1998). District laboratory Practise in Tropical Countries. Part 1.

[22] Midzi N, Mtapuri-Zinyowera S, Mapingure MP, Sangweme D, Chirehwa MT (2010). Consequences of polyparasitism on anaemia among primary school children in Zimbabwe.. Acta Trop.

[23] Hosmer DW, Lemenshow S (2000). Applied Logistic Regression.

[24] van der Werf MJ, de Vlas SJ, Brooker S, Looman CW, Nagelkerke NJ (2003). Quantification of clinical morbidity associated with schistosome infection in sub-Saharan Africa.. Acta Trop.

[25] Raso G, N'Goran EK, Toty A, Luginbuhl A, Adjoua CA (2004). Efficacy and side effects of praziquantel against *Schistosoma mansoni* in a community of western Cote d'Ivoire.. Trans R Soc Trop Med Hyg.

[26] Stelma FF, Talla I, Sow S, Kongs A, Niang M (1995). Efficacy and side effects of praziquantel in an epidemic focus of *Schistosoma mansoni*.. Am J Trop Med Hyg.

[27] Olds GR, King C, Hewlett J, Olveda R, Wu G (1999). Double-blind placebo-controlled study of concurrent administration of albendazole and praziquantel in schoolchildren with schistosomiasis and geohelminths.. J Infect Dis.

[28] Brindley P, Sher A (1987). The chemotherapeutic effect of praziquantel is against *Schistosoma mansoni* is dependent on host antibody response.. Journal of Immunology.

[29] Doenhoff M, Sabah AAA, Fletcher C, Weebe G, Bin J (1987). Evidence for an immune-dependent aaction of praziquantel on *Schistosoma mansoni* in mice.. Trans Royal Soc Trop Med Hyg.

[30] Butterworth AE, Curry AJ, Dunne DW, Fulford AJC, Kimani G (1994). Immunity and morbidity in human schistosomiasis mansoni.. Trop Geo Med.

[31] Gryseels B (1994). Human resistance to *Schistosoma* infections: age or experience.. Parasitol Today.

[32] Hagan P, Blumenthal U, Chaudri M, Greenwood B, Hayes R (1987). Resistance to reinfection with *Schistosoma haematobium* in Gambian children: analysis of their immune responses.. Trans Royal Soc Trop Med Hyg.

[33] Hagan P, Blumenthal UJ, Dunne D, Simpson AJG, Wilkins AH (1991). Human IgE, IgG4 and resistance to reinfection with *Schistosoma haematobium*.. Nature.

[34] Mutapi F, Ndhlovu PD, Hagan P, Spicer JT, Mduluza T (1998). Chemotherapy accelerates the development of acquired immune responses to *Schistosoma haematobium* infection.. J Infect Dis.

[35] Mutapi F, Hagan P, Ndhlovu P, Woolhouse MEJ (1997). Comparison of humoral responses to *Schistosoma haematobium* in areas with high and low levels of infection.. Parasite Immunol.

[36] Stelma FF (1997). Immuno-epidemiology, morbidity and chemotherapy in a community recently exposed to *Schistosoma mansoni* infection..

[37] Webster M, Correa-Oliveira R, Gazzinelli G, Viana IR, Fraga LA (1997). Factors affecting high and low human IgE responses to schistosome worm antigens in an area of Brazil endemic for *Schistosoma mansoni* and hookworm.. Am J Trop Med Hyg.

[38] Webster M, LibrandaRamirez BDL, Aligui GD, Olveda RM, Ouma JH (1997). The influence of sex and age on antibody isotype responses to *Schistosoma mansoni* and *Schistosoma japonicum* in human populations in Kenya and the Philippines.. Parasitology.

[39] Black CL, Muok EM, Mwinzi PN, Carter JM, Karanja DM (2010). Increases in Levels of Schistosome-Specific Immunoglobulin E and CD23(+) B Cells in a cohort of Kenyan children undergoing repeated treatment and reinfection with *Schistosoma mansoni*.. J Infect Dis.

[40] McMahon JE, Kolstrup N (1979). Praziquantel: a new schistosomicide against *Schistosoma haematobium*.. Br Med J.

[41] De Clercq D, Vercruysse J, Kongs A, Verle P, Dompnier JP (2002). Efficacy of artesunate and praziquantel in *Schistosoma haematobium* infected schoolchildren.. Acta Trop.

[42] Tchuente LA, Shaw DJ, Polla L, Cioli D, Vercruysse J (2004). Efficacy of praziquantel against *Schistosoma haematobium* infection in children.. Am J Trop Med Hyg.

